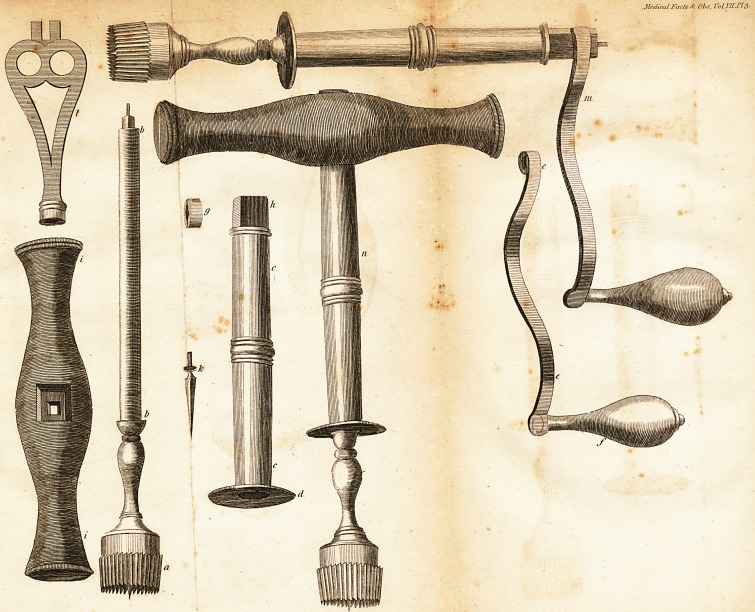# Description of an Instrument for Performing the Operation of Trepanning the Skull, with More Ease, Safety, and Expedition Than with Those Now in General Use

**Published:** 1797

**Authors:** Samuel Croker King

**Affiliations:** Member of the Royal College of Surgeons in Ireland, and M. R. I. A.


XVII. *Deſcription of an Inſtrument for performing the Operation of trepanning the Skull, with more Eaſe, Safety, and Expedition than with thoſe now in general Uſe. By* Samuel Croker King, *Eſq. Member of the Royal College of Surgeons in Ireland, and M. R. I. A.—From the Tranſactions of the Royal Iriſh Academy,* Vol. IV.

THE various accidents and diſeaſes which have ever been the lot of the human frame, muſt have called loudly on mankind to exert themſelves for the relief of their ſuffering fellow-creatures; from theſe incidental calamities, the different operations of ſurgery have taken their riſe, for the performance of which inſtruments judiciouſly contrived and properly conſtructed were abſolutely neceſſary, as well for the eaſe and ſafety of the patient, as for the dexterity of the operator; while ſuch were wanting, danger to the one, and difficulty to the other, unqueſtionably muſt have exiſted.

The improvements from time to time made in in the mechanic arts have afforded to the modern practitioners of ſurgery a manifeſt advantage over thoſe of earlier ages; ſo that I may venture to affirm, that by the conſtruction and workmanſhip of the inſtruments (joined to the aſſiſtance the operator derives from a knowledge of anatomy) more lives amongſt the objects of ſurgery are at this day ſaved than in times paſt in ſimilar caſes.

If we look into the works of the old writers on the ſcience of ſurgery, and examine the inſtructions handed down to us by them, for the performance of the principal operations, with the deſcriptions they have given us of the inſtruments they employed, we cannot wonder if many of their patients as often periſhed by the ill ſucceſs of their operations, as by the maladies they attempted to remedy or remove. Notwithſtanding (as Mr. Pott* juſtly obſerves) they were well acquainted with the neceſſity of operations, yet, wanting the aſſiſtance of the ingenious mechanic to form their inſtruments, their intentions of affording relief to their patients were frequently fruſtrated.

In the preſent century, though the mode of

* Pott's Works, Vol. I. page 122.

performing moſt of the capital operations in ſurgery, and the inſtruments proper for each, have undergone very conſiderable improvements and alterations, yet there ſeems to be room left for purſuing this uſeful branch of the healing art ſomewhat farther, particularly in the operation of trepanning, or perforating the cranium, which has continued to be effected with inſtruments fabricated nearly in the ſame form for a ſeries of years; and conſidering that this operation muſt have been often neceſſary, from the accidents which have never ceaſed to occur, it is a matter of ſome ſurpriſe that the inconveniences attending the two inſtruments now generally uſed, namely, the trepan and the trephine, ſo called, have not been in ſome meaſure removed. The only attempt that has fallen under my obſervation was by Mr. John Douglas, a figure of whoſe inſtrument may be ſeen in the Edinburgh Medical Eſſays *, and thus deſcribed: "Two plates "of braſs, kept together by four pillars of "braſs, with a handle moving a tooth-wheel, "which turns a pinion to which the ſocket "for receiving a common ſaw-head of a tre-

* Vol. V. Table iv. Fig. 6. page 374.

" pan is fixed." This defcription is followed by a remark, " That the faw will be turned " *more equally* with this inftrument than with " the hand alone ; but whether the rattling and " trembling which the wheels make are fuffi- " cient to counterbalance this advantage, I " fhall not determine." By this we may fuppofe it had never been introduced into practice. To the above objection might be added, the chance that a machine of the conftruction now defcribed might be liable eafily to be put out of order: though the ingenuity of the invention muft be acknowledged, and no doubt Mr. Douglas was well fatisfied that fomething to render the operation of trepanning more expeditious, fafe, and eafy, was wanting.

Every furgeon will admit, that both the trepan and trephine are attended with inconveniences; as a proof of this, I need only produce the teftimonies of two of the lateft authors, who have enriched the fcience of furgery by their writings, namely, Mr. Pott, of London, and Mr. Bell, of Edinburgh. The former* having ftrongly recommended the trephine in

* Pott's Works, Vol. I. Note in page 125.

prefepreference to the trepan, and the latter *, on the contrary, having given his decifion in favour of the trepan. Thefe inftruments are too well known to require a minute defcription here. I fhall only remark, that the trepan is compofed of a circular faw called a crown, fixed in a handle, which is turned round like a joiner's brace, with a knob on the upper part, on which the left hand of the operator is refted, to keep the inftrument fteady, while the right is employed to turn the handle. The trephine has a faw or crown of the fame fort, fixed in an immoveable handle, either of wood or iron, fomewhat refembling the handle of a carpenter's auger, and is worked by turning it backward and forward with the right hand.

The inconveniences † of the trepan muft be obvious to every experienced operator, who will often find a difficulty to get either himfelf

* Bell's Syftem of Surgery, Vol. III. page 78.

† Quia Chirurgus trepanum fæpe non recte tenet, nec ortoganaliter ponit fuper cranium, ex quo fit ut uno latere tangat meningem antequam perforaverit alterum, hoc autem ex eo fæpe contingere poteft, quod oculus Chirurgi ex alto afpiciens non bene poffit videre an trepanum recte ftet, nec ne, nifi ab aftante medico admoneatur. Thomas Fienus de Trepano, Tractat. 1, cap. 3. fol. 6.

 or or his patient into a commodious ſituation. To operate with it, he muſt be placed above the patient, eſpecially if the perforation is to be made on or near the upper part of the cranium. The aſſiſtants muſt be uncommonly attentive to keep the patient's head very ſteady, for the leaſt motion will throw the trepan out of its direction, which, from its length, it is liable to. The difficulty of keeping a patient quiet, (unruly from the effects of the accident, or impatient under an operation which perhaps with reluctance he has ſubmitted to) is well known to every operator.

To remove thoſe impediments, ſome operators, beſides keeping one hand firmly preſſed on the knob, place their forehead on it, as directed by Dionis *, or their chin, as adviſed by Garengeot †; theſe ſituations, beſides very much confining the operator, muſt preclude him from ſeeing the progreſs of the ſaw; and when the furrow has been made into the ſecond table of the ſkull, and the reſiſtance againſt

* Dionis, Cours d'Operations de Chirurgie. 5eme. edit. 8vo. Paris, 1765, p. 520.

† Traité des Operations de Chirurgie, Tom. III. p. 187, 188. 8vo. Paris, 1731.

the the inſtrument is become feeble, the bone may give way, and the ſaw, by being ſuddenly preſſed in, may injure the dura-mater and brain, and conſequently death enſue.

To prevent accidents of this kind, the older ſurgeons guarded the ſaw with wings or ſhoulders, and Ambroiſe Paré * tells us, he invented a ferula or ring, which he applied to the ſaw, with a ſcrew to ſecure it from too ſuddenly entering through the ſkull; afterwards, when the wings, ſhoulders, and ferulæ were laid aſide, the ſaws were made of a pyramidal form, and were ſerrated on the ſides as well as at the edge.

Heiſter †, ſufficiently aware of the danger attending the trepan, adviſes, that when the ſkull is ſawed deep enough, which may be known by the circular piece being a little looſe, a terebra or gimlet ſhould be ſcrewed into the hole made by the center pin, and by the help of an elevator the piece is to be taken out; if it is not looſe enough to come away, a few more turns of the ſaw are to be made, and the

* Johnſon's Tranſlation of the works of Ambroſe Parey, Book 10, chap. 18, p. 245.

† Heiſter's Surgery, part. 2, chap. 41, page 361.

terebra applied again, to bring out the piece without hazarding the wounding the dura mater by too frequent applications of the ſaw. And in all authors who have treated on this operation, we may find abundant cautions againſt injuring the brain or its membranes, by a want of attention, when the ſkull is nearly perforated. Theſe conſiderations, no doubt, determined Mr. Cheſelden*, Mr. Sharpe†, Mr. Pott‡, and moſt of the eminent ſurgeons in England, to give the preference to the trephine, as a more ſafe and handy inſtrument.

The trephine has, in reſpect of ſafety, ſome advantage over the trepan; the operator can with more readineſs apply it to any part of the head; being ſhorter, it is more manageable; and as there is leſs occaſion for preſſure on it, there is conſequently leſs hazard of its ſuddenly ſlipping in and injuring the brain or its meninges; but then it is tedious, and divides the bone very ſlowly; the ſaw does only ſcrape the bone, the pronation and ſupination of the wriſt

* Cheſelden's Obſervations on Le Dran's Surgery, page 447.

† Sharpe on the Operations of Surgery. 8vo. 1769, p. 157.

‡ Pott's Works, Vol. I. page 123.

cannot cannot give it quite half a circle, and it is impoſſible to keep the hand ſo exact in its movement, but the furrow or ſulcus will be *very uneven*; in ſhort, it is not only fatiguing to the operator, but tireſome to the patient, eſpecially if more than one perforation is to be made, which ſometimes happens, as may be ſeen in Dionis*, when the operation of trepanning was repeated twelve times on a young woman who had fallen from a ladder, by Meſſrs. Mareſchal and Dionis, with their two ſons. Mr. Gooch † recites a caſe where thirteen perforations were made on the ſkull of an old man, who recovered. Scultetus ‡ has given a caſe in which he made ſeven perforations round a depreſſion, to diſengage the fractured piece; and a very eminent practitioner § of this city (while I am writing this) informs me, that very lately he made three perforations with the trephine, which he aſſures me was attended with

* Dionis, Cours d'Operations, page 522.

† Gooch's Caſes, Vol. II. 8vo. 1767, plate 1. page 1.

‡ Scultetus, Armament. Chir. Obſ. 5. 8vo. 1659, p. 198.

§ James Henthorn, Eſq. Secretary to the Royal College of Surgeons, in Ireland.

very great fatigue. If neceſſary, many like inſtances might be produced.

Though ſawing the bone does not amount to pain, yet no doubt while that work is going on a diſagreeable ſenſation to the patient muſt be excited; therefore, the more expeditiouſly it can be finiſhed, conſiſtently with ſafety, the better.

If theſe diſadvantages attend the uſe of the two modern inſtruments, what muſt have been the caſe in former days, when the terebra or ſcrew, the drill, the chiſel, and leaden mallet, to break off the uneven edges of a fracture, or to divide the ſpaces between the perforations made by the terebra, were in uſe? or the meningophylax, which was a flat piece of ſilver or copper, like a ſpatula, thruſt between the ſkull and the brain, to defend it from receiving injury from the chiſel, when ſtruck by the mallet of lead? Is it not charitable to conclude, that many of the patients of thoſe days were permitted to cloſe their exiſtence without the aid of gimlets, drills, mallets, or chiſels?

Mr. Pott *, in his Obſervations on Injuries of the Head, has given us a very particular

* Pott's Works, Vol. I. Note in page 125.

account account of the instruments used by the ancient surgeons. As his works are in the hands of every practitioner, I shall only observe, that he has rejected the trepan as an *unmanageable instrument*, "liable to most of the hazard and "inconvenience attending the terebræ and te- "rebellæ of the ancients." In his opinion he coincides with Mr. Sharpe, who, in his Treatise on Operations, gives the preference to the trephine in the following words: "I have used "the word trepan all along for the sake of be- "ing understood; but the instrument I recom- "mend is a trephine*;" the advantages of which he describes, in the reference to the plate, as deserving a preference to the trepan, which, he says, is the instrument used in all parts of Europe, except Great Britain. And Mr. Chefelden†, in his Observations on Le Dran's Operations, recommends the trephine in private operations, but the trepan when expedition is necessary, as in a battle or sea engagement. The handle of the trephine he desires to be made so heavy, that the hand may have little more to do than to direct it; but

* Sharpe's Surgery, p. 157.

† Chefelden's Observations on Le Dran, p. 447.

Mr.

Mr. Pott, differing in this particular from him, advifes* the handle to be made of light wood.

The inftrument delineated by Mr. Sharpe, and approved of by him, is an exact copy of that recommended by John Woodall, Surgeon to St. Bartholomew's Hofpital in London, and Surgeon General of the Eaft India Company, in the reign of King Charles the Firft, in a fecond part of a work of his, entitled, "The "Surgeon's Mate, or Military and Domeftique "Surgery;" he gives a plate and a long defcription of an inftrument of his own invention, which he calls a *trafine*, (a tribus finibus) it being made with a handle, that each extremity might ferve the purpofe of an elevator of a different form; thus combining three inftruments in one. The edition of the work now before me was printed in the year 1639, but I find, from the preface, he had written it in the year 1626, when in Italy: and as his defcription nearly corrrefponds with the inftruments recommended by Mr. Chefelden and Mr. Sharpe, it is probable, on his recommendation, it had before their time been admitted into practice. My

* Pott's Works, Vol. I. Note in page 123.

late friend, the celebrated Dr. M'Bride *, has borne teſtimony, that John Woodall was a man of knowledge, experience, and obſervation; and, as a proof, refers us to his accurate account of the ſymptoms of the ſea ſcurvy, and his directions for the treatment of that dreadful diſorder, ſo often fatal in long voyages. He has beſtowed many pages in enumerating the advantages of his trafine, and the diſadvantages of the trepan, which, with all its faults, muſt have been deemed an acquiſition, when invented, to the ſcience of ſurgery; at a time when the terebra, terebella, abaptiſta, mediolus, meningophylax, mallets, chiſels, &c. were the inſtruments in common uſe, no doubt the trepan muſt have thrown all thoſe into diſrepute. Fallopius † (among others) has condemned the mallet and chiſel, and has cautioned his readers againſt them, leſt by-ſtanders and the public ſhould, if the patient died, attribute his death to the treatment of the operator.

* M'Bride's Experimental Eſſays, Eſſay 4, p. 186, 209.

† Ideo nolite uti hujuſmodi ſcalpro, cum malleolis, ſed potius manibus, ſi vulgus veſtras eſt ita ut noſtras. Fallopii Opera. Folio. Francofurti, 1606. Tom. I. p. 579.

We

We find even the writers of times not ſo remote, cautious of recommending the operation of trepanning. Peter Lowe, one of the firſt Engliſh writers on ſurgery, who ſtyles himſelf Doctor in the Faculty of Chyrurgerie at Paris, and ordinary Chyrurgion to the French King and Navarre, in his work called, "A "Diſcourſe of the whole Art of Chyrurgery," printed in London, in the year 1634, treating on the operation of trepanning, has theſe remarkable words: "There is great judgement "to be uſed in doing this operation, and few "there are found that doe it well; many I "have ſeen of very learned and expert men, "and heard of divers to my great joy and "comfort, among which Mr. George Baker, "ſome time Chyrurgion Ordinarie to that wor- "thy Prince* Queen Elizabeth, and now to "his moſt ſacred Majeſtie; a man of great "learning and experience, moſt fortunate and "dexter in this operation, like as in all other "operations of Chyrurgery;" and concluding his chapter with this pious ejaculation, "*God* "*increaſe the number of ſuch in this kingdome,*" plainly indicates that, at the time he wrote,

* So in the original, which is in black letter, p. 320.

the the ſcience of ſurgery had not arrived to ſo flouriſhing a ſtate in Britain as it has in theſe days. And John Woodall, the inventor of the traſine, in a former part of his work deſires the young practitioner not to proceed too haſtily in the uſe of the trepan, " for," ſays he, " many* worthy artiſts there are at this day " living, which have performed great cures in " fractures of the cranium, and yet never " knew the worthy uſe of this inſtrument," (the trepan); and then he tells us that, in eight years living in Germany, he could not find that the German ſurgeons uſed a trepan, though they both ſpoke and wrote about it.

The Engliſh tranſlator † of the works of Ambroiſe Paré has given us a figure of the traſine, and has quoted for his authority Doctor Helkiah Crooke, a phyſician, who flouriſhed in England in the reign of King Charles the Firſt. Probably the Doctor took the hint from John Woodall. By the dates of their works they appear to have been cotemporaries. In an

* Woodall's Military and Domeſtique Surgery, page 4.

† Johnſon's Tranſlation of the works of Ambroſe Parey, book x. chap. 18, page 246.

appenappendix * to his Microcoſmographia, he gives the figures of three-and-fifty inſtruments of chirurgery; after that of the trepan, (which, with all the others, is copied from Ambroiſe Paré, as acknowledged in his title page) he gives a figure of another ſort of trepan or traſine, as in general uſe then amongſt the London chirurgeons; it exactly reſembles that of John Woodall, and is thus deſcribed, as called the head trepan: "The head of which is "made taper faſhion, ſmaller at the teeth, "and greater upwards, with cutting edges "round about on the outſide, to make way "for itſelf; the ſhank of the head entereth "into the ſocket of a ſtraight ſtemme, and is "made faſt into it with a ſcrew; the handle is "made croſs the top of the ſtemme, like the "handle of a gimlet, but larger, and both "ſides made in the form of an elevatory; this, "he ſays, with a ſemi-circular motion of the "hand, performeth the operation with great "ſecurity, for the perforation being made *it* "*cannot ſlip in, to endanger the hurting the dura* "*mater, as the other may do.*"

* Appendix to Crooke's Microcoſmographia, chap. 17, page 25.

In tracing the progreſs of the two inſtruments now in uſe for perforating the ſkull, we find that the traſine was introduced into practice in England in the laſt century, and ſucceeded to the trepan in its improved ſtate; and though for ſome years paſt the trephine has been generally uſed by the Engliſh ſurgeons, yet, as I mentioned before, Mr. Bell has given a decided preference to the trepan, and has, by his recommendation, endeavoured to recal it into practice. His words are, "* If the trephine is employed, all the *preſſure and force* "neceſſary for turning the inſtrument is applied by one hand of the operator; the ſaw "is made to cut by forming half a circle only, "or ſcarcely ſo much, and the perforation is "finiſhed by moving the ſaw backward and "forward, till the whole thickneſs of the bone "is divided; but when the trepan is made uſe "of, the ſurgeon applies all the *preſſure upon* "*the head of the inſtrument with one hand*, "while he turns the handle of it with the "other. Some operators indeed make the "preſſure upon it with their forehead, or with "their chin, but it is more eaſily and more

* Bell's Syſtem of Surgery, Vol. III. page 77.

"equally

" equally applied with one hand, than it possi-

" bly can be in any other manner; by the tre-

" pan the saw is made to move always in the

" same direction, by which it cuts more easily,

" and performs the operation in one-third of the

" time that is necessary with the trephine; as it

" often happens that several perforations are

" necessary, and as the operation is of conse-

" quence *fatiguing to the operator and distressing*

" *to the patient, that method of operating ought*

" *surely to be preferred which renders the opera-*

" *tion more easy, provided it is at the same time*

" *equally safe.*" After having given this decision in favour of the trepan, for expedition and safety, he has taken notice, " that some prac-

" titioners, very sensible of these advantages

" of the trepan, but dreading the risk *of its*

" *passing too suddenly in upon the brain*, com-

" mence the operation with this instrument,

" and finish it with the trephine. This, he

" says, is far preferable to the usual method of

" performing the operation entirely with the

" trephine; but those who have fully experi-

" rienced the advantages of the trepan will

" employ it for the whole operation."

I have been particular in reciting this passage, as Mr. Bell (to whom the science of surgery gery is much indebted) has so widely differed in his choice of the instruments for perforating the skull, from the opinion of Mr. Cheselden, Mr. Sharpe, and Mr. Pott; but if we examine candidly the objections of Mr. Bell to the trephine, (some of which are certainly well-founded) we shall find they may, with equal justice, be applied to the trepan. If force or pressure be necessary for the trephine, force and pressure must surely be necessary for the trepan; without a degree of pressure the instrument cannot be kept in its place; for when the center pin is removed, is not the part of the bone within the sulcus the center round which the saw must turn? And if some sort of pressure be not made on the knob, by the sustaining hand, the saw cannot be retained in the furrow, so as to cut equally, but will, with the least motion of the patient's head, be thrown out, by which the operation must be retarded. The instrument, by reason of its length (few of them being less than eleven inches from the knob to the teeth of the saw, many of them more) cannot be replaced very expeditiously; it must be admitted, that it works quicker and more equably than the trephine; but though the trafine invented by John Woodall was made

 to to cut in its motions backward and forward, (that is, I ſuppoſe, the teeth of the ſaw ſet alternately in oppoſite directions) yet notwithſtanding this mechaniſm, that it is laborious and tedious every experienced ſurgeon will allow; no doubt this was the reaſon which induced many operators to begin the operation with the trepan for expedition, and finiſh it with the trephine for ſafety.

In the hands of a judicious and careful operator, who has had opportunities of frequently practiſing this operation, either of the inſtruments now in queſtion might be ſafe, and I ſhould be ſorry to ſuppoſe that any fatal accidents have happened in theſe our days in conſequence of uſing them without due attention. But when we conſider that trepanning is an operation of the utmoſt neceſſity, and ſeldom will admit of delay; that the patient who has met the accident, and is to be relieved, is often too remote from ſuch help as he or his friends would wiſh to apply to; that the neareſt practitioner is to be called upon, who muſt go to work without the advantage of having the advice or aſſiſtance of an aſſociate of the profeſſion, which is not the caſe in general with moſt other ſurgical operations, where the patient has time to consider and make choice of the person into whose hands he will commit himself, and leisure perhaps to remove to the place where the assistance he approves of is to be obtained; but injuries of the head will not at all times allow of such advantages.

When, I say, we consider all these circumstances, and reflect that so many of the most eminent and able operators have differed materially in the choice and form of the instruments for the purpose of perforating the cranium, and that the objections made to any one instrument were in some measure applicable to them all, we shall be led to think, that if an instrument could be devised, in which might be united the expeditious and equal working of the trepan, with the safety of the trephine, a valuable addition thereby would be made to the manual part of surgery: It will be readily granted, that in every profession all are not alike expert in using their hands; this may be observed in every mechanical trade or occupation; but if we can render the instruments so simple in their structure, that all difficulty in working will be removed, we shall thereby bring the performers more on an equality; and as in the operation we have been treating of, a cautious attention to avoid injuring the brain or its membranes is fo requifite, we cannot be too ferioufly on our guard. Though the bufinefs of trepanning, fimply confidered, is no more than fawing a portion of the fkull, yet that bufinefs, injudicioufly or incautioufly executed, may be the caufe of putting an end to the life intended to be faved, or as John Woodall expreffes it, * " A man may in a mo-" ment be flaine by art, for want of art."

In ufing either the trepan or trephine, force or preffure, for obvious reafons, fhould, as much as poffible, be avoided. We may obferve that a carpenter, in fawing, knows, that if he applies too much weight on his faw he will retard its progrefs; therefore he depends more on the dexterous manner of handling the faw than on his ftrength or the weight of his hand; and in thefe operations of furgery, which are merely mechanical, we fhould not difdain to take inftructions from the performance of thofe artifts from whom we have condefcended, in fome meafure, to borrow the fafhion of our inftruments. The truth of what I have advanced every furgeon muft have perceived

* Page 317.

when ſawing through the bone in amputating, and a little experience will teach him, that he will ſooner accompliſh the work by a proper adroitneſs than by dint of force or ſtrength.

The inſtrument which I have the honour of laying before the Academy, I am inclined to think, will render that neceſſary operation of trepanning more eaſy, more expeditious, and more ſafe. If I have ſucceeded in this attempt, I ſhall eſteem myſelf fortunate to have contributed to the improvement of ſo uſeful an art as that of ſurgery; but if I have done no more, I have offered another inſtrument, to thoſe already in uſe, for the operator to chooſe from, in an operation which requires to be properly and cautiouſly performed, and is frequently the chief, ſometimes the *only relief* for the perſon who has the misfortune to be the ſubject of it. In uſing this inſtrument, neither force nor preſſure is requiſite; the left hand, which is employed to ſuſtain the inſtrument, by being placed near the ſaw or crown, will keep it ſteady and firm to the place intended to be perforated, while the right hand is engaged in turning the handle faſter or ſlower, as the operator ſhall judge expedient; the points on which the ſaw turns being at each end of the canula, and not reſting on the two extremities of the inſtrument, (which is one of the faults of the trepan) will require no more force or preſſure on it, while working, than what may be ſufficient to keep the teeth of the ſaw in contact with the bone; conſequently, (preſſure not being neceſſary) all hazard of ſuddenly entering or wounding the dura-mater or the brain is prevented.

This inſtrument is compoſed of a crown or ſaw made in the uſual form, and about an inch and an half from the crown is fixed to a ſpindle four inches and an half long, which is received into a barrel or canula of four inches in length; to the top of the ſpindle, which is ſquare above the canula, is applied a handle or winch, with a nut ſcrewed on the ſpindle to keep the handle on; at the lower part of the canula or barrel is a flat rim, projecting about a quarter of an inch, on which the left hand, which graſps the canula, reſts, to prevent it from ſlipping down on the part of the inſtrument below it, which is turned by the handle above; the crown has a center-pin, as in the other inſtruments, with a key to remove it when the ſulcus is deep enough to admit it to be taken away. Though with this the operation may with great

ſafety ease and safety be entirely completed, yet to accommodate those who wish to finish with the trephine, the upper part of the barrel or canula is made square to fit into a wooden handle; upon applying this handle, instead of the winch, the instrument is converted into a trephine; in this wooden handle is a square opening, fitted to the square part of the spindle, and fastened by the same nut.

It will be requisite to have two or three crowns of different sizes, that the operator may choose that which will best suit the circumstances of the case; as to the form of the crowns, modern practitioners have so differed about them, that every surgeon must be left to his own choice. For my part, I should prefer those as the best and safest which are neither too conical nor too cylindrical, but between both extremes; and, as Mr. Cheselden * has advised, the cavity in the inside to enlarge in the same manner the outside does, to prevent the piece of bone to be taken out from being wedged in the cavity, and to allow the crown or saw to be inclined to one side or to the other, as occasion may require, during the operation.

* Cheselden's Observations on Le Dran, page 447.

That the form of this inſtrument may be better underſtood, I have annexed engraved figures *, not only of the parts of it ſeparately; but likewiſe of all theſe parts together, when to be uſed.---I am well aware, that in offering this inſtrument to the public, I may have to combat not only with the prejudice of a great many years, but with ſome of the moſt reſpectable authorities in the profeſſion, who have been long in the habit of uſing the other inſtruments with ſucceſs; but from the experience I have had of it myſelf, and from the approbation it has met with from many eminent ſurgeons in this city. I am tempted to ſend it into the world, with a hope that no practitioner will condemn it without firſt having given it a fair trial; if, then, any alteration ſhould be ſuggeſted, to render it more ſafe, uſeful, and convenient, the improvement ſhall be thankfully adopted.

I muſt obſerve, that in the foregoing pages the reader will find the inſtrument called trephine ſometimes written *traſine*. In this I have followed the ſpelling of the authors I have conſulted. Trafine, a *tribus finibus*, was the name

* See Plate III.

l
b
b
g
h
i
c
c
k
b
c
d
i
a
n
m
e
e
f it received from the inventor, John Woodall; it has ſince, for what reaſon I know not, been changed to trephine; but the alteration in the letters of the word (the pronunciation being the ſame) is immaterial.

### *Explanation of the Plate* *.

*a* The crown or ſaw.

*bb* The ſpindle to which the ſaw is faſtened. Note, the ſpindle is to be made ſmaller from each end to the middle, that it may not touch the barrel but at the upper and lower parts, to prevent too much friction in turning.

*cc* The canula or barrel into which the ſpindle is introduced.

*d* The lower part of the canula, which projects ſomething above a quarter of an inch, on which the left hand is reſted.

*ee* The handle, with a ſquare opening to fit the end of the ſpindle.

* The drawings for this plate were taken by Mr. John Ellis, from the firſt-made inſtrument, which was executed with great exactneſs, from my verbal inſtructions, by Mr. John Read, an eminent cutler and inſtrument maker, of Skinner Row, in this city.

*f* The

*f* The knob of the handle, which ſhould be made of ivory.

*g* The nut to ſcrew on the end of the ſpindle to keep the handle on.

*h* The end of the canula, formed ſquare to fit the wooden handle when uſed as a trephine.

*ii* The wooden handle fitted to receive the canula and ſpindle; when uſed as a trephine, the upper ſide is counter-ſunk to admit the nut.

*k* The center-pin of the crown or ſaw.

*l* The key, one end of which is made to fit the center-pin, and the other, which is forked, to fit the cavity in the top of the nut.

*m* A view of the inſtrument, with the ſeveral parts put together when uſed as a trepan.

*n* A view of the inſtrument when intended for a trephine.

## Figures and Tables

**Figure f1:**